# Identifying barriers to care for complex airway disease and multidisciplinary solutions to optimize therapy in Canada

**DOI:** 10.1186/s40463-022-00576-8

**Published:** 2022-04-15

**Authors:** A. Cherukupalli, M. Yong, Y. Chan, M. Desrosiers, A. Thamboo

**Affiliations:** 1 Division of Otolaryngology-Head & Neck Surgery, Department of Surgery, Diamond Healthcare Center, University of British Columbia, 4th Floor, 2775 Laurel St., Vancouver, BC V5Z 1M9 Canada; 2 Department of Otolaryngology-Head & Neck Surgery, Department of Surgery, University of Toronto, Toronto, ON Canada; 3 Division of Otolaryngology-Head & Neck Surgery, Department of Surgery, McGill University, Montreal, QC Canada

**Keywords:** Multidisciplinary, Complex airway disease, AERD, Chronic Rhinosinusitis, Cross-disciplinary, Allergy, Respirology, Rhinology

## Abstract

**Background:**

Complex airway disease such as Chronic Rhinosinusitis with Asthma or Aspirin Exacerbated Respiratory Disease requires a multidisciplinary approach to management and treatment. Many centers in the USA have created collaborative multidisciplinary clinics to support the management of these patients; however, similar structures do not appear to exist in Canada.

**Methods:**

This mixed methods study used a combination of structured interviews and a cross-sectional national survey. Interviewees included members of the Canadian Rhinology Working Group and survey participants were a combination of academic and community Rhinologists, Respirologists and Allergists. All participation was voluntary and selection criteria was based on their involvement in treating complex airway disease. Our objective was to identify the current state of diagnosis and treatment of complex airway patients in Canada between Rhinology, Respirology and Allergy and understand the barriers, challenges and propose solutions to establishing a multidisciplinary airway clinic in Canada.

**Results:**

Four Rhinologists participated in qualitative interviews and a convenience sample of 42 specialists through our known network responded to our quantitative survey. From our survey, 54.8% believed multidisciplinary clinics were necessary in the management of complex airway disease, providing better outcomes and cost-savings (69%, 45.2%). Most specialties agreed that history, physical, pulmonary function and skin prick testing was important for diagnosis (92.9%, 92.9%, 88.1%). If clinicians were to participate in a multidisciplinary clinic, they would be willing to forego an average of 14.2% of their mean daily income for that clinic. The ideal clinic location was split between a neutral shared location vs. a Rhinology clinic space (38.1%, 45.2%).

**Conclusions:**

Complex airway diseases are currently managed in subspecialty silos resulting in fragmented care. Our study highlights gaps in management, areas for improvement and support for establishing multidisciplinary complex airway disease clinics in Canada to better treat this population.

## Background

Complex airway disease consists of many subdivisions including but not limited to Chronic Rhinosinusitis (CRS) with/without polyposis, bronchial asthma, and Aspirin Exacerbated Respiratory Disease (AERD). CRS affects approximately 12.5% of the US population with AERD affecting 0.3–0.9% with a higher prevalence in asthmatic patients (3–20%) [1, 2]. The current treatment algorithm for many of these conditions involves a combination of medical and surgical management [1]. However, being multi-system diseases, different specialists have different approaches to both diagnosis and respective treatment algorithms. This can sometimes cause redundancy in the patients’ diagnostic workup. Ultimately, their proposed treatment may not be optimized for their particular disease process. [3, 4]. This lack of consensus for diagnostic evaluation and treatment between specialists due to siloed care negatively impacts patient satisfaction, quality of life and drives up healthcare costs [3].

The increasingly-recognized importance of multidisciplinary collaboration when diagnosing and treating complex diseases has led to advances in the organization and delivery of healthcare [4]. Multidisciplinary clinics (MDC) have proven to be beneficial in overall patient satisfaction and outcomes in a variety of settings. For example, MDCs have shown reduced mortality rates in cancer patients and improved quality of life and control levels among asthmatic patients with a reduced number of exacerbations [5, 6]. Several clinics in the USA have created Complex Airway MDCs. These centers have multiple subspecialists in their staff including Rhinologists and Allergist/Immunologists that facilitate holistic care for complex airway patients through a single, coordinated team [7, 8].

Although there is evidence of complex airway MDCs in the USA, a similar system does not appear to be well-established in Canada thus, limiting the treatment of these patients to individual providers, specifically Otolaryngologists and Rhinologists, due to their surgical expertise. However, Rhinologists often lack the specialized training required to provide certain therapeutic interventions such as aspirin desensitization therapy, immunomodulatory therapy or asthma management. Therefore, these patients are often neglected from such therapies, or are not referred to the appropriate Respirologist/Allergist in a timely manner due to the need for referral to a specialist at a separate clinic. These limitations place a cap on the ability of clinicians to optimize the care provided to complex airway patients in Canada. The purpose of our study is to identify the current state of treatment of complex airway patients in Canada, as well as understand the barriers and challenges to establishing complex airway MDCs in Canada. Also, we plan to highlight a pragmatic outline for the establishment of MDCs in Canada.

## Methods

A mixed methods study was employed involving structured narrative interviews, as well as an anonymous cross-sectional survey distributed to both community and academic Rhinologists, Allergists and Respirologists across Canada from March 20, 2021 to May 20, 2021. Approval for the study was granted by the University of Toronto’s Research Ethics Board (Protocol#: 00040369).

### Narrative interviews

An initial literature review searching Pubmed between the dates of 2000 and 2021 was performed using the keywords “multidisciplinary”, “AERD”, “chronic rhinosinusitis”, “asthma”, and “allergy”. A series of closed and open-ended interview questions were created around themes such as: complex airway disease diagnosis and management, specialty referral patterns and perceived barriers and/or thoughts about complex airway MDCs (“Appendix”). Members of the Canadian Rhinology Working Group were interviewed in a semi-structured format by the principal investigator to ensure consistency. Interviewees were selected based on a convenience sample available to the principal investigator.

### Interview thematic analysis

There were 2 main themes that emerged from the narrative interviews, and these were: (1) The necessity for a MDC, (2) The logistical burden involved is a major hindrance to implementation. All participants agreed that MDC would be essential to improving complex airway disease care and other subspecialists are interested in collaboration. One individual mentioned they could also be “modelled off of skull base clinics or similar to tumor boards” that have already established success with multidisciplinary collaboration. Several interviewees however identified “different fee schedules for different specialists, clinic location and available equipment” as potential barriers to implementation. A clinical coordinator and clinic space were recurrent priorities among interviewees for the successful establishment of a MDC.

### Anonymized survey

Using the qualitative data gathered from the narrative interviews, an anonymized survey was created by the study authors and distributed using the UBC Qualtrics Survey tool. Questions from the survey were based on the themes that were identified during the interviews including complex airway diagnosis, MDC interest and frequency, as well as perceived benefits for establishment of MDC in Canada. The survey was completely voluntary and distributed to a convenience sample of both community and academic Rhinologists within the Canadian Rhinology Working Group who were asked to distribute the survey to Allergists and Respirologists they share patients with for the management of complex airway patients in their practice.

Statistical analysis was conducted using the extracted data in the form of descriptive statistics. No further rigorous statistical analyses were performed. Additionally, common themes were analyzed surrounding MDC establishment in Canada through open text in the survey and interview responses.

## Results

### Demographics

A total of 4 members of the Canadian Rhinology Working Group were interviewed as part of the qualitative aspect of the study. A total of 42 participants responded to the online survey. These respondents included academic and community Rhinologists (n = 17, n = 5), academic and community Respirologists (n = 8, n = 2) and academic and community Allergists (n = 6, n = 4). Of all respondents, 74% had an academic practice, 26.2% had a community practice, 59.5% mainly practiced in a hospital setting and 40.5% in a private clinic (Table 1).

**Table 1:** Demographics

	Total (n)
*Specialty*	
Rhinology	22
Respirology	10
Allergy/immunology	10
*Location*	
Western Canada	13
Eastern Canada	24
Unanswered	5
*Years in practice*	
≤ 5	12
6–9	4
≥ 10	26
*Practice type*	
Academic	31
Community	11
*Location of practice*	
Hospital	25
Private Clinic	17
*Group/solo practice*	
Group	16
Solo	26

### Diagnostic criteria and management

In the diagnosis of upper airway disease, most participants ranked history and physical examination as the most important. The majority of Rhinologists believed nasal endoscopy was an important diagnostic tool; however, the necessity of this assessment was not as strongly reflected by Respirologists and Allergists, where only 70% and 80% respectively deemed it necessary. With regards to lower airway disease, most participants agreed in the importance of history and physical examination. Respirologists noted that methacholine challenge and Pulmonary Function Tests (PFT) were the next important diagnostic investigations (100%, 90%). In contrast, just over half of Rhinologists felt that a methacholine challenge test was a necessary investigation in diagnosing lower airway disease (59%). Similarly, with allergy determination, Allergists felt history and skin prick testing were the most important diagnostic tests. There was a wide variety of responses from the Respirologists and Rhinologists (Tables 2, 3, 4).

**Table 2:** How participants diagnose upper airway disease

Method	Rhinology	Resp	Allergy
History (%)	20 (91%)	9 (90%)	10 (100%)
Physical exam (%)	20 (91%)	8 (80%)	9 (90%)
CT scan (%)	16 (73%)	10 (100%)	9 (90%)
Nasal endoscopy (%)	18 (82%)	7 (70%)	8 (80%)

**Table 3:** How participants diagnose lower airway disease

Method	Rhinology	Resp	Allergy
History (%)	19 (86%)	10 (100%)	10 (100%)
Physical exam (%)	19 (86%)	7 (70%)	10 (100%)
Pulmonary function tests (%)	20 (91%)	9(90%)	10 (100%)
Methacholine challenge test (%)	13 (59%)	10 (100%)	8 (80%)

**Table 4:** How participants diagnose allergy

Method	Rhinology	Resp	Allergy
History (%)	18 (82%)	9 (90%)	10 (100%)
Physical exam (%)	14 (64%)	8 (80%)	9 (90%)
Skin prick testing (%)	18 (82%)	9 (90%)	10 (100%)
Intradermal skin testing (%)	8 (36%)	3 (30%)	6 (60%)
Spirometry (%)	6 (27%)	1 (10%)	5 (50%)
IgE specific testing (%)	12 (55%)	8 (80%)	9 (90%)
Oral challenge test (%)	8 (36%)	7 (70%)	9 (90%)
Patch testing (%)	4 (18%)	5 (50%)	5 (50%)

This variability in prioritization of diagnostic testing amongst specialists may influence the referral patterns between the different specialties. Therefore, some patients may not receive the necessary diagnostic workup depending where they fall within the algorithm and therein not be provided with the best treatment for their specific disease.

### MDC interest, structure and cost

Most participants felt that MDCs provide better care for complex airway disease (69%) and should be established in Canada (54.8%). Of all the potential airway pathologies to be referred to a Complex Airway MDC, most participants agreed that CRS with asthma (88.1%) was the most appropriate, with other possible diagnostic referrals including AERD (83.3%) and Cystic Fibrosis (64.3%) (Table 5). Regarding frequency, most respondents felt that a Complex Airway MDC should be held monthly (54.8%).

**Table 5:** MDC diagnostic referrals

Diagnosis	Rhinology	Resp	Allergy
Chronic rhinosinusitis with asthma (%)	19 (86%)	9 (90%)	9 (90%)
Isolated uncontrolled upper airway disease (%)	6 (27%)	2 (20%)	3 (30%)
Isolated uncontrolled lower airway disease (%)	5 (23%)	2 (20%)	3 (30%)
Cystic fibrosis (%)	17 (77%)	3 (30%)	7 (70%)
AERD (%)	18 (82%)	8 (80%)	9 (90%)
Other (%)	3 (14%)	3 (30%)	1 (10%)
Unanswered (%)	0 (0%)	1 (10%)	0 (0%)

The ideal location of the clinic was evaluated based on the equipment necessary for each specialty to diagnose airway disease. Most Rhinologists believed that the clinic should be held at a Rhinology clinic location (63.64%). However, averaged across specialties, a neutral zone appeared to be the agreed-upon ideal clinic location (Table 6). Regarding the income loss associated with seeing fewer patients per day in a MDC, all participants agreed they would be willing to sacrifice a mean income of 14.2% during that clinic day to run this clinic.

**Table 6:** MDC clinic location

Location	Rhinology	Resp	Allergy
Neutral zone (%)	7 (32%)	2 (20%)	7 (70%)
Rhinology (%)	14 (64%)	3 (30%)	2 (20%)
Respirology (%)	0 (0%)	3 (30%)	0 (0%)
Allergy/immunology (%)	1 (5%)	1 (10%)	1 (10%)
Unanswered (%)	0 (0%)	1 (10%)	0 (0%)

## Discussion

Complex Airway MDCs have been well established in the USA, which have demonstrated significant improvement in quality of life indicators and symptom recurrence [8]. However, a lack of similar clinics in Canada has limited the treatment potential of complex airway disease patients in this country. Our study evaluated the current diagnostic and treatment algorithms of Rhinology, Respirology and Allergy in Canada, as well as interest in and barriers to establishing a Complex Airway MDC in Canada.

The need for a MDC is based on the shared pathophysiology between the sinonasal cavity and the lungs. For example, CRS with polyps has a Type II inflammatory pattern characterized by eosinophilia and elevated IL-4, 5 and 13 cytokines [9]. Lower respiratory tract manifestations are characterized by a similar systemic inflammatory response [10]. Approximately 60% of patients with CRS with polyps have lower airway disease including co-existing asthma [9]. Treatment of the upper airway can modify the severity of lower airway disease and vice versa, and early treatment may also help prevent further progression of the patient’s airway disease [10]. However, to achieve this, sub-specialists are required to collaborate in the diagnosis and management of these complex airway diseases to provide appropriate specialized treatments. Establishing a MDC will facilitate this interaction, allow patients to receive proper diagnostic workup and tailored treatments from the relevant sub-specialists on the team. While it is true that multidisciplinary meetings such as case conferences provide avenues for similar collaborative care, we believe that gathering providers in a physical MDC space provides value both for patients, who can attend one appointment instead of many, and for providers, who can share diagnostic findings such as endoscopic examinations in real time.

Although there was expressed interest in establishing a MDC (69%), we identified 4 fundamental barriers through our interviews: Current Dogma, Control, Location and Funding. In our current system, sub-specialists operate independently and practice through a blend of personal experience and evidence-based medicine. Challenging this status quo and asking practitioners to forego some degree of independence to optimize patient care through evidence-based medicine may initially be difficult. There is a similar barrier with regards to the preferred location of the MDC. Finally, to maintain both research and clinical pillars, the MDC would require a steady stream of funding. Any loss in income flow because of lower patient volumes in a MDC may disincentivize clinicians from participating, as well as restrict research resource availability.

**Fig. 1:**
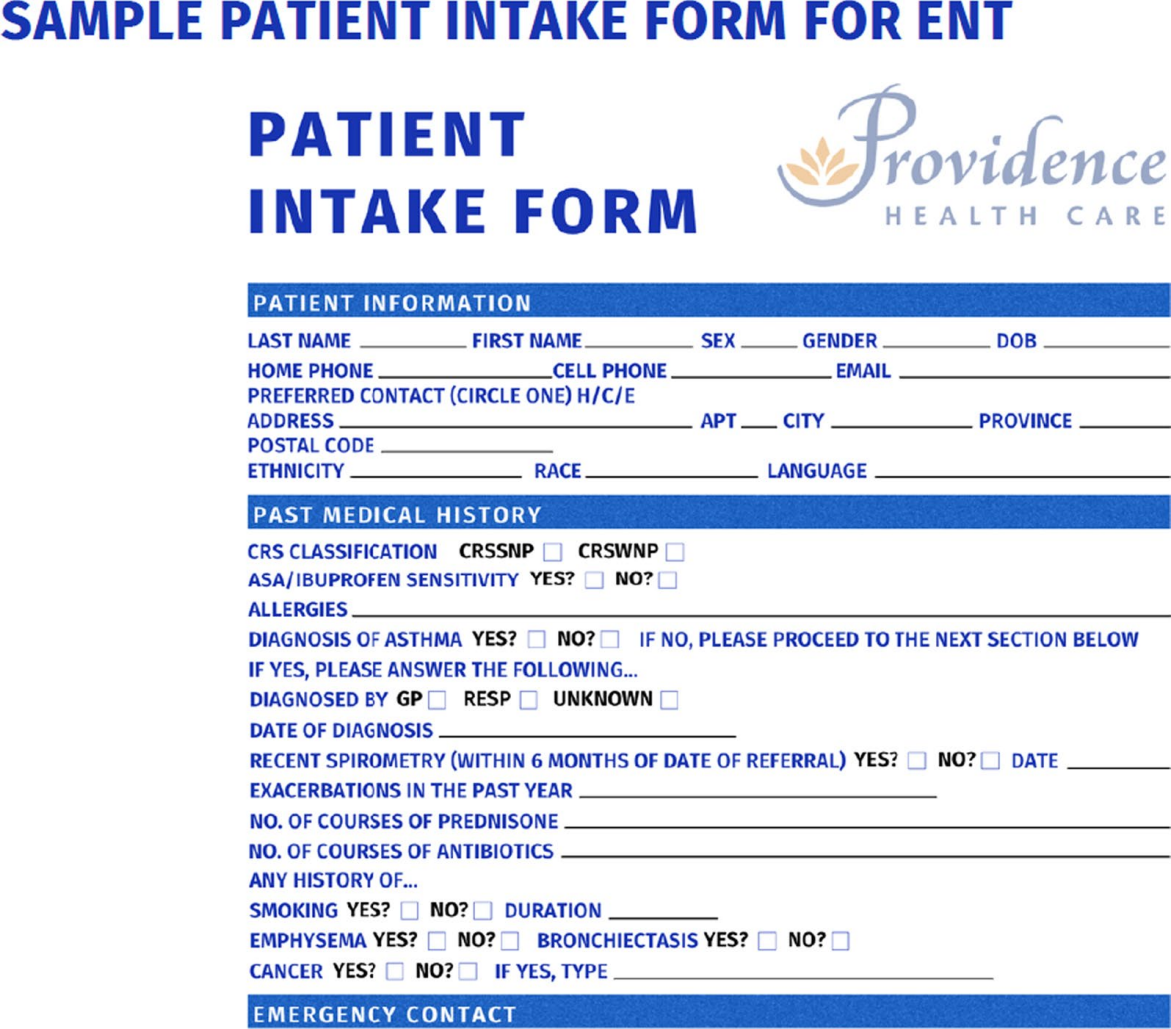
Rhinology patient intake form

**Fig. 2:**
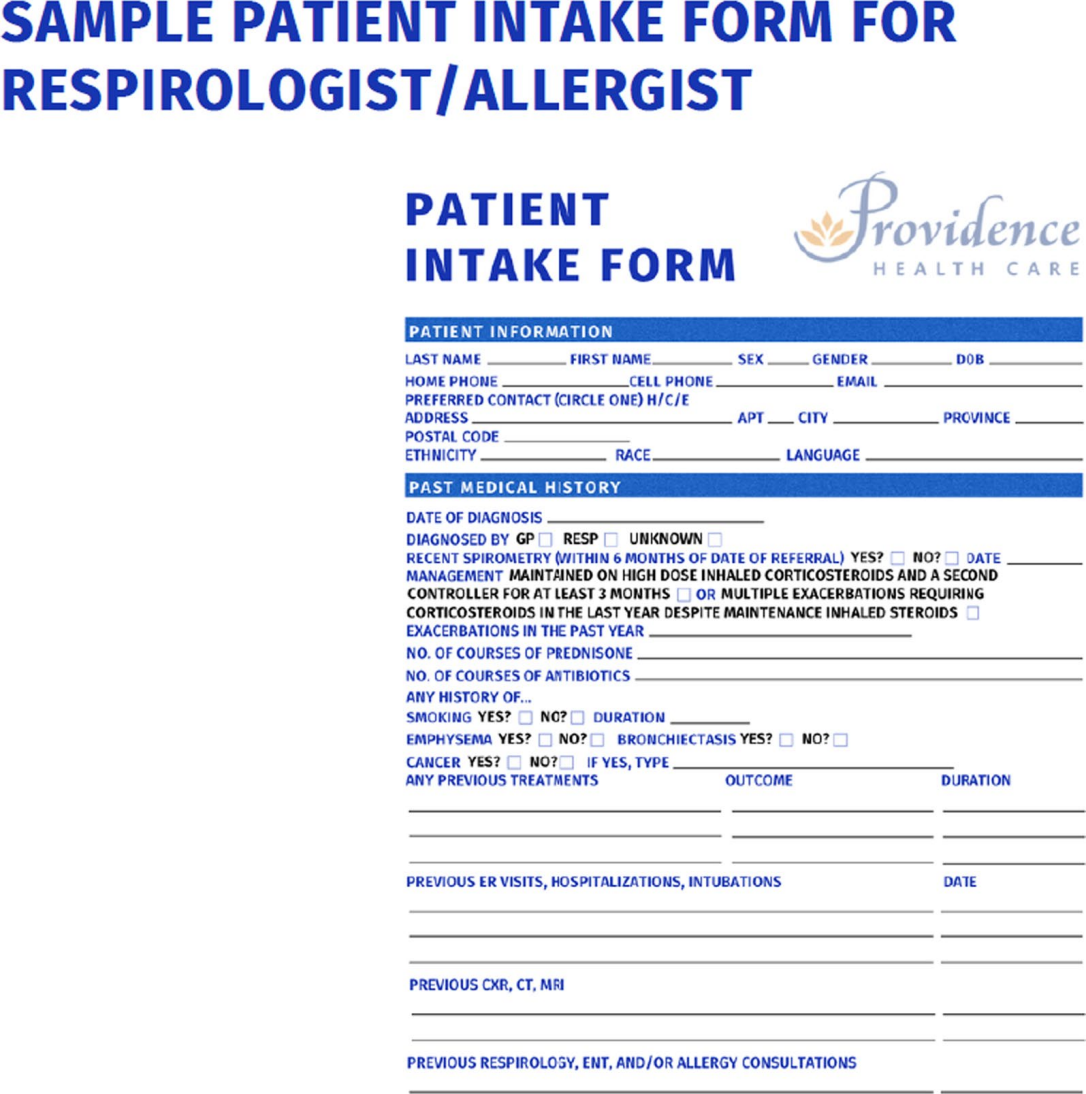
Respirology and allergy patient intake form

To address these barriers, we have identified a roadmap to the development of a Complex Airway MDC in Canada. First, is the creation of a team of subspeciality leaders from Rhinology, Respirology and Allergy. Each specialty must identify a lead individual to represent their interests in putting the MDC together. This is particularly important in larger centres where they may have multiple subspecialists working at the same site. As outlined from the results of this study, there is a gap in understanding how to best manage upper and lower airway disease. The management algorithm for patients defined with complex airway disease requires consensus among each specialty. Once a defined population and management algorithm is finalized, the team must determine: (1) Who is the referral group? (i.e., primary care, specialists) and (2) What intake form will be used by the referring physician to screen for appropriateness. At the University of British Columbia (UBC), a MDC has been established and the referral group was defined as Otolaryngologists, Respirologists and Allergists. The defined referral base limits the incoming referrals to consist of only complex patients with most diagnostic testing already complete. For example, a patients with AERD would already have PFTs supporting a diagnosis of asthma, a documented allergy to aspirin, and a diagnosis of CRS based on such guidelines as EPOS 2020 (clinical symptoms in keeping with CRS and either endoscopic evidence or CT imaging findings of mucosal changes). For patients with CF, sweat chloride testing would already be done along with possible genetic screening. Otolaryngologists have a different intake form compared to Respirologist and Allergists, as the skillset of each specialty is different (Figs. 1 and 2).

**Fig. 3:**
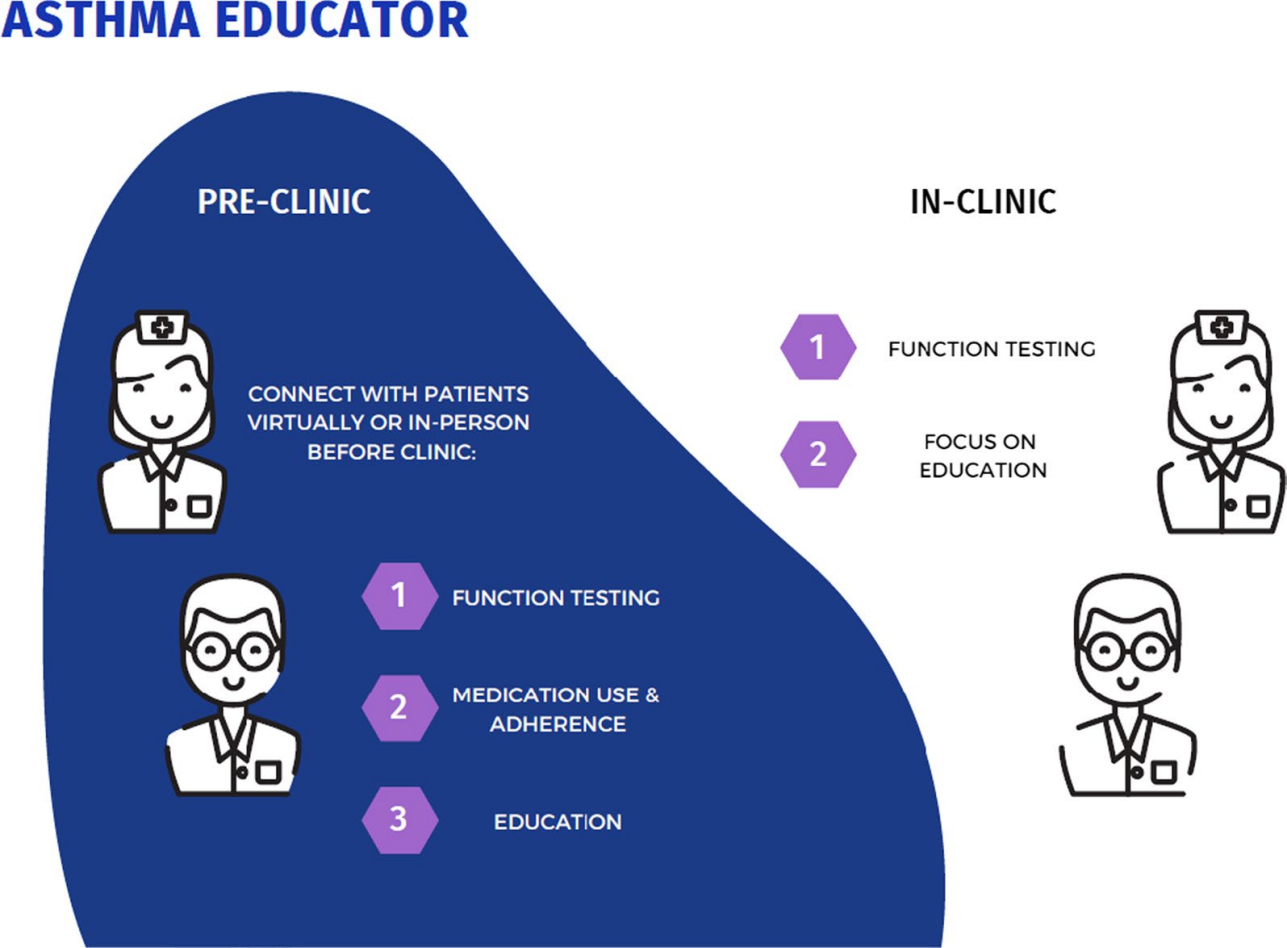
Asthma educator role

**Fig. 4:**
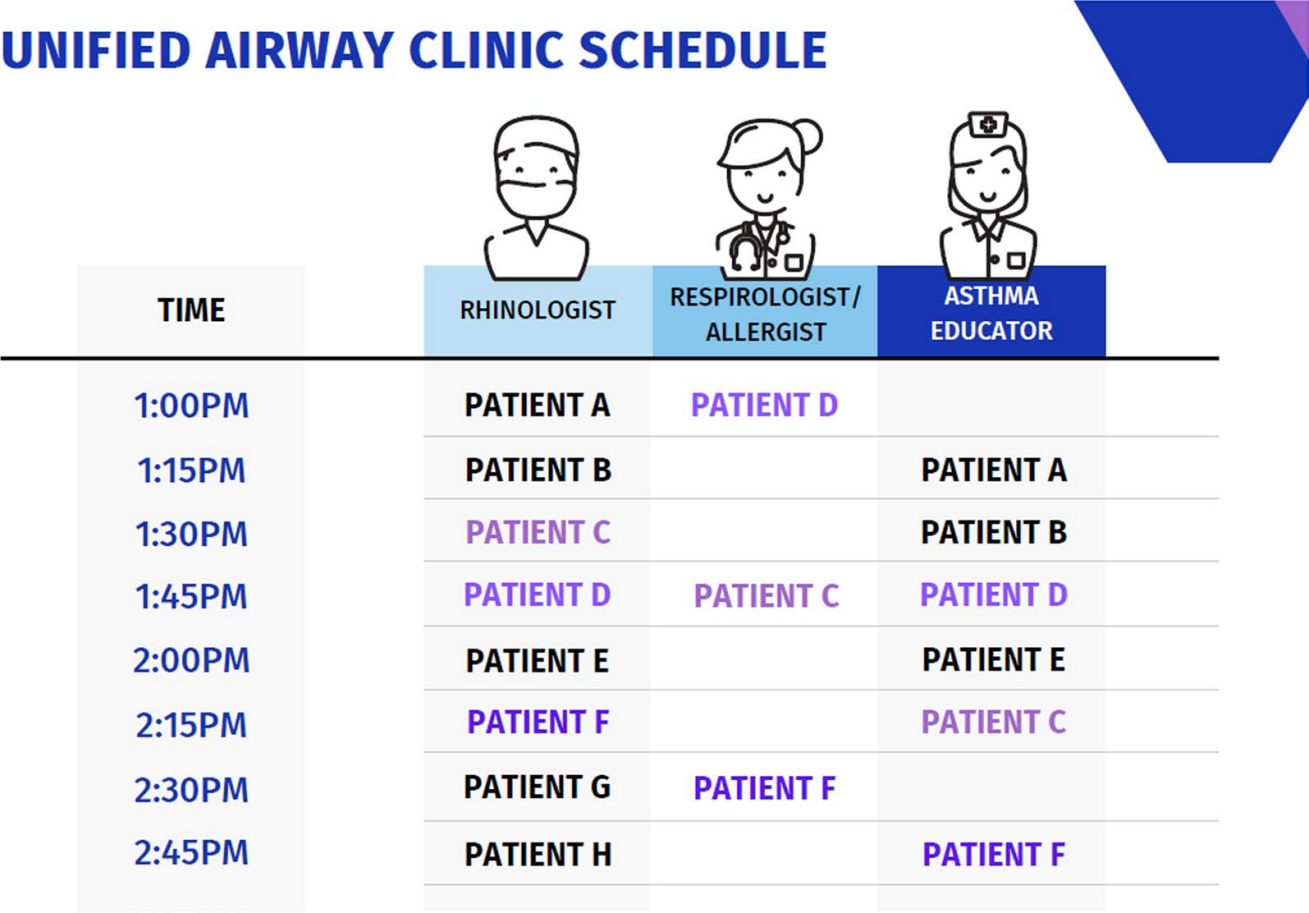
Sample clinic schedule

Moreover, for the MDC to be successful, the leads of the MDC must define the resources required and what resources are already available. Rather than requesting resources from the health authority/hospital, re-allocation of already available resources was more economical. Common resources used by Respirologists are asthma educators/technicians who provide education, pulmonary function testing, and allergy testing. Re-allocation of this role into the MDC provides a lot of value to the patients (Fig. 3). Another important resource is the clinical coordinator to coordinate the administrative steps between initial referral, timing specialist assessment in clinic and education. A sample patient clinic schedule has been provided to outline the logistical coordination involved (Fig. 4). Furthermore, individual administrative assistants and coordinators at other MDCs in the hospital could also be reallocated for assistant in clinic logistics.

Another possibility is the combination of a clinical/research coordinator role, as the nature of the MDC lends itself well to providing opportunities for research. At UBC, the MDC is viewed as a research clinic and re-allocation of research funds between the three specialties to pay for a research coordinator has been a successful strategy. This helps to manage the clinic and optimize research productivity, which is an effective strategy to circumvent the lack of funding from the health authority and/or hospital to financially support the clinic. Furthermore, with a focus on the research pillar of the clinic, the complex airway MDC also acts as a center for excellence for the development of predictive medicine, markers, and cutting-edge evidence-based treatment strategies.

Most participants in our study would prefer a ‘neutral-based’ clinic location. However, at UBC, we have found that the biggest issue is the capital required to support rhinology equipment, which includes endoscopes, video towers and the support staff required to clean the scopes. Given that the infrastructure is already in place in Rhinology clinics, the MDC was brought to the Rhinology clinic at our institution. In this setup, portable pulmonary function testing and allergy kits were easier to implement into the Rhinology clinic than creating more expensive alternative arrangements. In addition, given the rapid advancement and adoption of telemedicine technology during the COVID-19 pandemic, adding teleconsult and videoconferencing capabilities to MDCs could allow for more versatile inclusion of asthma educators and consulting providers who do not need to conduct any additional in-person diagnostic testing. This could help the establishment of MDCs in community and more rural areas outside of tertiary care academic centers as long as the necessary rhinology, pulmonary, and allergy equipment are available when needed.

Overall, the majority of the decision making and operationalization of the MDC was built around a business model where no further funding was requested. Instead, we relied on a re-allocation of existing resources. This required all three involved specialties to come to the table providing an equal share to the MDC. We have summarized our recommendations for establishing a MDC in the attached figure (Table 7). Above all, cooperative leadership and open communication plays a strong role in the success of MDC.

**Table 7:** Key points to setting up a MDC Airway Clinic

*Strategy to creation of a MDC Airway Clinic in Canada*
Creation of team of subspeciality leaders in Rhinology, Respirology and Allergy
Determine the referral group and intake form for the clinic
Define resources required: consider re-allocation of already available resources (ex. Asthma educators)
Obtain an effective clinic coordinator to help with operations as well as in a research coordinator capacity
Establish a mutually agreed upon and economical clinic space
Creation of a business model without the need for additional funding based on cooperative leadership and open communication

An additional point of importance after setting up the MDC is how to keep community colleagues involved in the care of these patients. It is our belief that a MDC in an tertiary care center would serve as an important initial touchpoint for patients with complex disease in order to facilitate access to medications such as biologics in a targeted and cost-effective manner. After optimal treatment plans are formed within the MDC, some of these patients could be followed on an ongoing basis by individual community providers and referred back to the MDC as needed.

Major limitations of our study include the sample bias inherent with the method of interviewing and survey distribution. A convenience sample of providers within the principal investigator’s professional network was used to gain access to second degree connections representing an array of Respirologists and Allergists. This sampling method may have, therefore, missed a large cohort of Otolaryngologists, Respirologists/Pulmonologists, and Allergists who may have had differing opinions. However, our results and thematic analysis are consistent with existing literature highlighting the barriers to effectively providing multidisciplinary care in the setting of complex diseases (11, 12). In addition, it may be difficult to reproduce the findings of our study in other healthcare systems outside of North America due to differences in healthcare provider compensation and public vs private insurance coverage.

## Conclusion

Complex airway disease in Canada is currently managed in sub-specialty silos resulting in limited health outcomes for patients. Our study highlighted both diagnostic and therapeutic discrepancies, as well as an interest and acknowledged benefit of establishing a complex airway MDC in Canada. Overcoming barriers to its establishment is possible and can be done through a shared decision-making model of subspecialty physicians poised to revolutionize the care of these patients in Canada.

## Data Availability

The datasets generated and/or analyzed during the current study are not publicly available due to participant privacy and are on an encrypted drive but are available from the corresponding author on reasonable request.

## Appendix: Interview Questions

**Name:**

**Position:**

**City:**

*Medical Management:*

**General thoughts about Aspirin Desensitization Therapy:**

- What are your thoughts about Aspirin Desensitization Therapy (ADT)?
- How often are you referring patients for ADT?
- Do you know of anyone in your city that has been providing or accepting referrals for ADT?

**Understanding of Literature around ADT use:**

- What is your understanding of the literature surrounding ADT in AERD patients?
- How has the current evidence about ADT affected your practice and management of AERD patients?

**Evaluating thoughts and barriers to establishing MDCs in Canada:**

- Is there a multidisciplinary AERD clinic in your city/province?
- What barriers prevent optimal management of your AERD patients (ex. Hospital infrastructure, physician interest, etc.)? If you had a magic wand, what would you change?
- How does access or lack of access to ADT affect you?
- Are other sub-specialists (Allergy/Immunology, Respirology) interested in collaborating with you to treat AERD patients?
- Are physicians compensated for ADT in your province? If so, how?
- What incentives do you believe would need to be in place for physicians to increase their willingness to offer ADT?

**Identifying the role of biologics in complex airway disease:**

- What do you believe is the role of biologics in AERD?
- Do you have easy access to prescribing biologics as a rhinologist?
- Where does biologic use fit into your treatment algorithm? In relation to ADT?
- Would you prefer to use a biologic or ADT following initial surgery (post-primary surgery)? Why?

**Other:**

- If ASA desensitization is being done, how do people do it? What is the protocol? Who is involved in this process?
- Where are these AERD patients being referred from to your practice?

*Surgical Management:*

**Approaches to surgical management of AERD:**

- How do you surgically manage AERD patients that present to your clinic without any prior surgery?
- How does your surgical management change with recurrent disease?
- Do you follow a particular algorithm in how you surgically manage AERD patients? What information helps inform your decision-making in particular treatment options?
- In primary AERD cases, what types of surgical procedures do you offer your patients?
- When would you perform extended surgical procedures in the management of AERD (i.e. more extensive than Draf IIa, MT resection, etc.)?

Is there anything I missed about the themes today that you would like to add to or comment on?
